# The vascular endothelium as decision maker in lung injury

**DOI:** 10.3389/fcell.2025.1564627

**Published:** 2025-07-07

**Authors:** Diana Klein

**Affiliations:** Institute of Cell Biology (Cancer Research), University Hospital, University of Duisburg-Essen, Essen, Germany

**Keywords:** vascular dysfunction, damage, endothelial activation, pulmonary endothelium, lung injury, inflammation

## Abstract

The vascular endothelium is the largest organ in the human body, capable of performing a wide range of cellular signaling and synthetic functions. It is also subjected to considerable mechanical stress due to the shear forces generated by blood flow, which amounts to approximately 10,000 L per day. The endothelial layer plays a crucial role in regulating vascular tone locally and controlling the extravasation of certain blood components. Additionally, it is integral to the coagulation process. The endothelium serves as the entry point for immune cells, which migrate from the bloodstream to surrounding tissues by passing through the endothelial layer. This underscores the importance of proper endothelial function for the health of the body’s tissues and organs. When the endothelium fails to perform these functions adequately, leading to endothelial dysfunction, pathological conditions are more likely to develop. Notably, acute lung injury and its severe form, acute respiratory distress syndrome (ARDS), are often associated with endothelial dysfunction. ARDS refers to pulmonary edema with increased vascular permeability resulting from various pulmonary or systemic insults. In most cases, an exaggerated inflammatory and pro-thrombotic response to the initial insult causes disruption of the alveolar–capillary membrane and leakage of vascular fluid. This review emphasizes the central role of the vascular endothelium in acute conditions and seeks to clarify the concepts and interplay between endothelial activation, dysfunction, and damage.

## Introduction

In a normal, healthy state, the endothelium is the innermost layer of blood vessels and is considered quiescent; that is, it maintains a state in which endothelial cells are resting, awaiting stimulation and activation signals. However, it is now clear that even a quiescent endothelium is highly active, and this state must be actively regulated (Ricard et al., 2021). The overall characteristics of healthy endothelial cells were recently summarized in an elegant review (Augustin and Koh, 2024), with the “life cycle” characteristics encompassing the mechanisms of vasculogenesis, angiogenesis, maintenance and quiescence during adulthood, and changes during aging. Further key characteristics of endothelial cells include their responsive and relaying functions, such as barrier function, cell trafficking control, perfusion permeability, and blood pressure regulation, as well as triggering coagulation. Additionally, they perform instructive functions such as mechanotransduction and metabolic regulation, particularly in an organ-specific manner (Augustin and Koh, 2024). In contrast, endothelial cells are critically involved in various life-threatening and chronic diseases, including cancer (De Palma and Hanahan, 2024; Klein, 2018), arterial diseases (e.g., cardiovascular disease, atherosclerosis, and hypertension) (Gallo et al., 2021; Xu et al., 2021; Wang and He, 2020), coagulation, and inflammation (van Hinsbergh, 2012), as well as in cerebral disorders (Sashindranath and Nandurkar, 2021) and aging (Franceschi et al., 2018). Thus, it has become clear that the vascular endothelium should be more widely appreciated as a systemically distributed organ with vital regulatory roles and instructive gatekeeper functions crucial for the maintenance of organ and system homeostasis (Augustin and Koh, 2024; Aug et al., 2017). A disruption of endothelial integrity and thus of endothelial barrier function represents the most striking pathophysiological change in acute lung injury (ALI), a life-threatening inflammatory lung disease usually caused by local or systemic inflammation and, in its most severe form, clinically manifesting as acute respiratory distress syndrome (ARDS) (Orfanos et al., 2006; Maniatis et al., 2008). Damage to the alveolar endothelium is accompanied by impairment of the alveolar epithelium, resulting in an overall dysfunction of the alveolar-capillary membrane barriers, which allows the exudation of protein-rich fluid into the alveolar space ultimately leading to pulmonary edema (Gu et al., 2024; Zhao et al., 2017). In addition, endothelial inflammation following endothelial activation leads to increased leukocyte adhesion, platelet aggregation and coagulation activation, which in turn can lead to endothelial cell damage and cell death, as well as microthrombosis and fibrin deposition further increasing vascular permeability (endothelial dysfunction). Here, the prevailing current concepts regarding the pulmonary endothelium are presented and an attempt is made to shed more light on the concepts and interactions between endothelial activation, dysfunction and damage.

## The pulmonary endothelium

The pulmonary circulation is unique among all vascular beds, because it receives all of the cardiac output while maintaining low vascular pressures (Hough et al., 2024). In combination with the formation of sufficiently thin endothelial-epithelial barriers, the blood-air barrier, this enables the main function of the lungs, the gas exchange (Mora Massad et al., 2025). Generally, three primary endothelial cell types are generally distinguished in lungs: pulmonary microvascular, pulmonary artery, and pulmonary vein endothelial cells (Przysinda et al., 2020; Aird, 2007). But the lung vascular bed is predominantly made up of microvascular endothelium, which facilitates the gas exchange between the bloodstream on the apical side and the air in the alveoli on the basal side (Amersfoort et al., 2022). And particularly pulmonary capillary endothelial cells harbor unique properties, which are the large numbers of caveolae, the expression of activated leukocyte cell adhesion molecule (ALCAM, also known as CD166) and the clotting factor (factor VIII) together with and the high expression levels of Angiotensin I–converting enzyme (ACE) (Aird, 2007) as well as the overall low permeability (compared to pulmonary arteries) (Aird, 2007; Qiao and Bhattacharya, 1985).

Among the capillary endothelium, two distinct endothelial subpopulations have been identified: aerocytes and general capillary endothelial cells, with aerocytes being rarer in the human lung than general capillary endothelial cells (Schupp et al., 2021; Sikkema et al., 2023; Travaglini et al., 2020). Aerocytes are described as expansive and intimately associated with type 1 pneumocytes. This capillary cell type exhibits a specialized gene repertoire, reflecting their roles in gas exchange and leukocyte trafficking, while the intermingled general capillary cells show the most significant changes in inflammatory signaling and stress responses in pulmonary diseases (Gillich et al., 2020). General capillary endothelial cells were shown to interact with aerocytes along the capillary axis and expressing N-cadherin, which forms heterotypic adhesions with surrounding pericytes. This capillary cell type is specialized to regulate vasomotor tone and may function as stem/progenitor cell in capillary homeostasis and repair (Gillich et al., 2020; Trimm and Red-Horse, 2023). The advent of single-cell analysis technologies has enabled further specification and identification of distinct endothelial subpopulations within the given organ and provided insights into how these cells respond to inflammatory stimuli (Zhang et al., 2022; Niethamer et al., 2020; Schupp et al., 2021). Carbonic anhydrase four-positive aerocytes in the alveolus, for example, display an atypically large “Swiss cheese”-like morphology, spreading over the thin alveolar type 1 epithelium. These cells receive reparative signals from alveolar type I cells, particularly in damaged alveoli during ALI (Niethamer et al., 2020). Single-cell RNA sequencing of lung endothelial cells, obtained after (lipopolysaccharide-induced) inflammatory lung injury, identified two major subpopulations within the lung microvascular endothelium. One subpopulation was characterized by enriched expression of immune response genes, such as MHC genes (designated as immune endothelial cells), and the other by increased expression of vascular development genes, such as Sox17, designated as developmental endothelial cells. The former exhibited a strong propensity for inflammatory signaling, while the latter showed a greater tendency for endothelial regeneration (Zhang et al., 2022). Both endothelial cell types appeared to align with the previously identified aerocyte and general capillary cell phenotypes. Angiocrine signals derived from the endothelium influence the fate of other lung progenitor cells, suggesting that pulmonary capillary endothelial cells instruct neoalveologenesis to restore gas exchange function in regenerating lungs, at least to some extent (Rafii et al., 2016). Consequently, the high regenerative potential of the lung microvasculature facilitates the efficient replacement of damaged or lost endothelial cells, contributing to the maintenance of (though impaired) vascular homeostasis following exposure to endothelial toxins or other environmental stresses (Taha et al., 2017). Furthermore, blood vessels harbor niches of endothelial progenitor and stem cells, which play essential roles in vascular repair and regeneration (Ergun et al., 2011; Klein et al., 2010). Particularly in the lungs, highly proliferative resident microvascular endothelial progenitor cells, capable of reconstituting the entire proliferative hierarchy of pulmonary microvascular endothelial cells, have been identified (Alvarez et al., 2008). However, the functions of individual endothelial cells and the full extent of heterogeneity among all lung endothelial cells remain incompletely understood despite the discovery of new endothelial cell types. In a more systematic approach, human lung scRNA-seq data were analyzed, and endothelial cell populations were characterized through iterative clustering and subsequent differential expression analysis (Schupp et al., 2021). This study identified two previously indistinguishable endothelial populations: pulmonary venous endothelial cells localized in the lung parenchyma and systemic venous endothelial cells found in the airways and visceral pleura (Schupp et al., 2021).

In summary, pulmonary endothelial cells, due to their strategic location at the interface between the bloodstream and lung tissue, play a key role not only in optimizing gas exchange and controlling barrier integrity and function but also in regulating the pulmonary vascular tone. Inhaled air exposes the lungs—the first point of contact—to various pathogens and pollutants. The pulmonary endothelium functions as an active and dynamic receptor-effector tissue, responding to various chemical, physical, and mechanical stimuli by secreting the appropriate substances to maintain vasomotor balance and vascular tissue homeostasis. As a vital part of the respiratory system, changes in the pulmonary endothelium play a central role in the pathogenesis of both acute and chronic lung diseases, a role emphasized by the term “orchestra conductor in respiratory diseases” (Huertas et al., 2018). Lung inflammation and its consequences, such as ALI and ARDS, present particular challenges in pulmonary and intensive care medicine. Although most ALI cases lack definitive identification of causative pathogens, respiratory viruses, common Gram-negative or Gram-positive bacteria, and mycobacteria are known to cause acute respiratory infections (Long et al., 2022; Matthay et al., 2019). The central processes involve endothelial cell activation, endothelial dysfunction, and the potential loss of endothelial cells, which, in turn, exacerbate the impairment of the structural integrity of lung endothelial barriers.

## Endothelial quiescence in normal lung homeostasis

Endothelial quiescence, and thus proper endothelial barrier function, is maintained through restricted permeability of the interendothelial connections formed by the adhesive properties of proteins that constitute tight and adherens junctions. In tight junctions, the extracellular domains of occludins, claudins, and junctional adhesion molecules create close and very tight endothelial cell–cell adhesions. Organotypic and vessel type-specific variations in claudin-5 expression, for example, contribute to the maintenance of varying degrees of barrier function in continuous vascular beds (Richards et al., 2022). Adherens junctions are mediated by the transmembrane protein vascular endothelium cadherin (VE-CAD), which facilitates homophilic adhesion between neighboring endothelial cells. The intracellular domains of these connecting proteins provide junctional stability through their interaction with the actin cytoskeleton *via* α-, β-, and p120-catenin or zona occludens-1/2/3 proteins (Van et al., 2008). P120, a multidomain intracellular protein, which mediates various cellular functions, including stabilization of cell-cell transmembrane cadherin complexes, contributes to endothelial permeability changes through its association with Rho GTPase activating protein (p190RhoGAP) and Rac1, whereby Rac1 activation by p190RhoGAP results in a reduction of RhoA activity to counteract increased permeability (Tian et al., 2013). Junctional barriers are generally dynamic structures with varying baseline differences in the organization of endothelial cell–cell junction components and protein expression, depending on the vascular bed (Brandon et al., 2024). An interendothelial or paracellular crossing of the endothelium occurs through small intercellular spaces between adjacent cells, allowing restricted passage of macromolecules larger than 3 nm in diameter through interendothelial junctions, while permitting convective and diffusive transport of smaller molecules under 3 nm in diameter (Van et al., 2008; Simionescu et al., 1978).

## Immune cell recruitment requires endothelial activation

**FIGURE 1:**
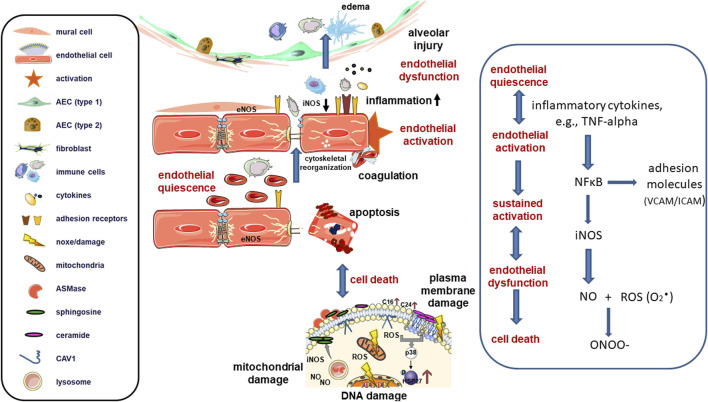
In a healthy state, mature endothelial cells (represented as red cells) are quiescent while performing a semi-permeable barrier function that regulates the supply of nutrients and oxygen to tissues and the removal of carbon dioxide and waste products. Nitric oxide (NO) produced through endothelial nitric oxide synthase (eNOS) is the primary regulator of blood flow and pressure. Upon activation, such as in response to pro-inflammatory cytokines and chemokines (e.g., TNF-alpha) or bacterial pathogens during lung infection or injury, inducible nitric oxide synthase (iNOS) becomes activated and generates high concentrations of NO through the activation of inducible nuclear factors, including NF-κB. Activated endothelial cells then upregulate cell adhesion molecules (selectins and integrins) to enhance leukocyte-endothelial cell interactions, facilitating the capture of circulating immune cells, such as neutrophils and monocytes, before extravasation into interstitial and alveolar spaces. Simultaneously, alterations in tight and adherens junctions allow excess extravasation of proteins, increasing endothelial permeability and leakage of bloodstream components. NO can reduce endothelial activation through inhibition of the transcription factor NF-κB and loss of NO increases endothelial cell activation. Endothelial activation can revert to its pre-inflammatory state, but prolonged/sustained activation can lead to irreversible endothelial dysfunction. Endothelial dysfunction in turn, leads to further increased permeability and transmigration of leukocytes through the endothelium, as well as to platelet activation and cytokine release. Together, these processes create a detrimental microenvironment, which damages the endothelial lining that can ultimately lead to endothelial cell death. Induced (sublethal) DNA damage and/or DNA repair deficiencies may also cause an activated endothelial state through activation of the genotoxic stress-induced NF-κB signaling pathway (“intrinsic” endothelial activation). In addition to endothelial DNA damage, the release of damage-associated molecular patterns (DAMPs) and the generation of reactive oxygen/nitrogen species (ROS/RNS) can further promote NF-κB activation. Superoxide (O_2_ **·**) as primary ROS/RNS species can rapidly react with NO to form peroxynitrite (ONOO−). Peroxynitrite in turn can cause multiple deleterious effects including tyrosine nitration of proteins and antioxidants inactivation finally mediating cell death and lung damage. Severe endothelial cell loss can occur as a long-term complication when the DNA-damaged and activated endothelial cells begin to proliferate. In most cases, endothelial cell death is apoptotic and involves the activation of acid sphingomyelinase (ASMase) and the subsequent formation of ceramides. In ALI, damage to both the vascular endothelium and alveolar epithelium occurs in response to systemic and local production of pro-inflammatory cytokines, resulting in the destruction of the endothelial and epithelial barriers in the lungs and the accumulation of edematous fluid and debris in the alveolar spaces.

Inflammatory processes depend on the ability of leukocytes to enter tissues from the bloodstream; this transendothelial migration of leukocytes, also known as leukodiapedesis, is essential for the immune response. An increased expression of immune-relevant adhesion molecules serves as a guide for leukocytes to the appropriate exit sites on the endothelium. This process is regulated by a coordinated action between endothelial cells and leukocytes, with endothelial cells activating leukocytes and guiding them to extravasation sites, which, in turn, instruct endothelial cells to create a path for transmigration (Vestweber, 2015). Lectin-like adhesion molecules, such as selectins, initially mediate the docking of leukocytes on the endothelial surface. Leukocytes slow down, allowing firm adhesion mediated by integrins and immunoglobulin-like adhesion molecules. This step initiates the regulated transmigration of the recruited cells, while simultaneously maintaining the integrity of the endothelial layer. Diapedesis of leukocytes mainly occurs intercellularly through the regulated temporary opening of interendothelial cell junctions; however, in certain cases, cells can also migrate transcellularly through individual endothelial cells. Prior to leukocyte transmigration, endothelial cells can extend membrane structures on their apical surface, forming clusters of endothelial adhesion receptors at exit sites, which are described as intracellular adhesion molecule I (ICAM-1)- and vascular cell adhesion molecule I (VCAM-1)-enriched “transmigratory cups” (Vestweber, 2015; Carman and Springer, 2004). The diapedesis process involves multiple functions of both leukocytes and endothelial cells, particularly the loosening of interendothelial cell contacts while preventing plasma leakage, active leukocyte migration through the junctional cleft, and sealing of the junction after diapedesis (Vestweber, 2015). These processes are not completely resolved in detail. The movement of leukocytes through the opened junctions between endothelial cells depends on adhesion molecules ICAM-1 and VCAM-1, which trigger tyrosine phosphorylation in VE-cadherin and/or component enrichment in platelet endothelial cell adhesion molecule 1 (PECAM-1), the diapedesis-mediating receptor CD99, and junctional adhesion molecules (JAM), particularly JAM-A. This may be supported by a multivesicular compartment, termed the lateral border recycling compartment (Vestweber, 2015; Muller, 2011; Mamdouh et al., 2003; Muller, 2014). Chronic exposure to inflammation further increases the endothelial production of matrix-degrading enzymes, such as matrix metalloproteinases (MMPs) and disintegrin and metalloproteinases (e.g., ADAM10), remodeling the basement membrane and facilitating leukocyte exit from the bloodstream into the tissue (Pruessmeyer et al., 2014). Increased intracellular calcium concentrations further contribute to increased vascular permeability following the activation of ADAM10, an extracellular sheddase that proteolytically cleaves VE-CAD, thereby reducing productive lateral endothelial contacts (Schulz et al., 2008). Increased cellular stress in general and particularly reactive oxygen species (ROS) production in endothelial cells are known to activate p38/MAPK signaling. Resulting (Rho/ROCK signaling-dependent) cytoskeletal (actin) rearrangements leading to the formation of stress fibers resulting in increased contractility caused retraction of junctional adhesion molecules (e.g., PECAM-1) away from the junction finally causing gaps between endothelial cells (Wang et al., 2024). ZO-1 and VE-cadherin can even be downregulated following p38/MAPK in the lung epithelium. Beside the dominating paracellular pathway, crossing the endothelium from the blood to the interstitium can also occur through the endothelial cell (also called transcytosis) as vesicle-mediated transport of macromolecules, e.g., plasma proteins, across the endothelial barrier in a caveolae-dependent manner (transcellular pathway) (Van et al., 2008). Increases in endothelial permeability were even linked to decreased expression levels of aquaporin 1, a protein involved in water and hydrogen peroxide balance through transcytosis, with accompanied increses in lung edema (Wang et al., 2024). Given the critical importance of continuously maintaining vascular homeostasis and integrity, hyperpermeability (vascular leakage) is associated with many pathological conditions. It can exaggerate disease severity and can lead to edema, reduced vascular perfusion, and finally impaired drug delivery (Figure 1).

## Endothelial activation

Immunomodulation by endothelial cells are of particular importance to ensure lung homeostasis and dampen immune activation following lung damage thereby promoting immunity (Amersfoort et al., 2022). It has long been established that inflammatory cytokines, such as interleukins (ILs) or tumor necrosis factors (TNFs), and bacterial products, such as Gram-negative endotoxins, modulate the adhesive properties of blood leukocytes in cultured human endothelial cells (Bevilacqua et al., 1985). Pathogen-associated molecular patterns (PAMPs) released from microorganisms during infections or damage-associated molecular patterns (DAMPs) released from injured tissue cells during tissue damage stimulate endothelial cells either directly, or even indirectly through activating cells of the innate immune system (such as mast cells, macrophages and dendritic cells), which then secrete cytokines and other pro-inflammatory mediators that in turn activate nearby endothelial cells of the microvasculature (Vestweber, 2015).

Endothelial activation represents a shift from quiescence and, thus, from immune surveillance to a phenotype that facilitates host defense reactions (inflammatory responses) (Klein, 2018). Endothelial activation is defined by the expression of cell-surface adhesion molecules following stimuli that facilitate the recruitment and attachment of circulating leukocytes to blood vessels. The upregulation of adhesive endothelial surface antigens includes human leukocyte antigen (HLA) molecules, particularly leukocyte adhesion molecules (e.g., E-selectin, ICAM-1/2, and VCAM-1). Additionally, other adhesion proteins have been identified in the endothelium, including immunoglobulin-containing and proline-rich receptor-1 (IGPR-1), and PECAM-1 (also known as CD31) (Wang et al., 2016). Beyond the upregulation of adhesive properties, endothelial activation also leads to an increase in the production of immuno-attractive cytokines (e.g., IL-6, IL-8, and MCP-1), while the loss of surface anticoagulant molecules, such as thrombomodulin and heparan sulfate induces pro-thrombotic changes in endothelial cells and/or alterations in vascular tone (e.g., deregulated production and/or bioavailability of nitric oxide, NO). Therefore, five core changes have been defined for endothelial cell activation: loss of vascular integrity, expression of leukocyte adhesion molecules, a shift in phenotype from antithrombotic to pro-thrombotic, cytokine production, and upregulation of HLA (Hunt and Jurd, 1998).

Primary inflammatory cytokines typically include TNFs and ILs, released from resident memory T and sentinel cells, such as mast cells, macrophages, and dendritic cells (DCs), to attract circulating blood to sites of tissue injury and infection. The primary goal of this process is to mitigate the initial inflammatory trigger and initiate tissue repair (Wang and He, 2020; Long et al., 2022). Similarly, inflammation-related and recruited white blood cells, including mast cells, are an important source of cytokines that affect the endothelium, including vascular endothelial growth factor (VEGF), leukotrienes, prostaglandins, cathepsins, interleukins, tryptases, platelet-activating factor, TNF-alpha, and other growth factors (e.g., FGF2, TGF-beta), which are released following secretion or from dying cells (Kunder et al., 2011; Giri et al., 2024). These substances, in turn, affect the local endothelium and inflammation and may foster systemic inflammation by delivering inflammatory factors into the blood circulation. Among the proinflammatory cytokines, TNF-alpha is one of the best known pleiotropic cytokines, whereby elevated concentrations under pathophysiological conditions induce an accumulation of inflammatory cells, stimulate the production of inflammatory mediators and can cause oxidative and nitrosative stress (Mukhopadhyay et al., 2006; Malaviya et al., 2017). TNF-alpha is mainly synthesized by macrophages and monocytes and exerts its effect *via* tumor necrosis factor receptors that can subsequently activate NF-κB/MAPK-mediated inflammation and/or induce apoptosis in the course of various complex formations (Mannel and Echtenacher, 1999). TNF-alpha mediated inflammation promotion in endothelial cells includes upregulation of adhesion molecules that are important for leukocyte transport to sites of inflammation as well as stimulation the release of eicosanoids, platelet-activating factor and other vasoconstrictors (e.g., endothelin-1), which in turn promote inflammation further through vasodilation, adhesion and migration of leukocytes (Malaviya et al., 2017). At the same time, TNF-alpha increases the permeability, which is based on endothelial barrier disruption *via* destabilization of adherens and tight junctions finally leading to pulmonary edema and hypoxemia (Ng et al., 2025). In addition to these activating cytokines, PAMPs, DAMPs, ROS, reactive nitogen species (RNS) and oxidized low-density lipoproteins (LDL) are known to directly and indirectly activate endothelial cells (Long et al., 2022; Liao, 2013). Beside these numerous biochemical stimuli, biomechanical stimuli (e.g., pulsatile blood flow, fluid shear stress, hydrostatic pressure, and cyclic stretching), can foster altered gene expressions in the endothelium finally causing endothelial activation (Gimbrone et al., 1997).

Endothelial activation can also result from indirect mechanisms, such as increased cellular stress and subsequent paracrine signaling from neighboring cells, including epithelial cells, fibroblasts, or infiltrating immune cells, or in response to genotoxic stressors. For example, genotoxic stress induced by radiation treatment in the lungs leads to senescence of the respiratory epithelium, a central process for the initiation and progression of lung diseases, particularly pneumonitis and fibrosis, both of which are strongly associated with endothelial dysfunction (Hansel et al., 2021; Hansel et al., 2020; Klein et al., 2017; Wiesemann et al., 2019). The pro-inflammatory and pro-oxidative senescence-associated secretory phenotype (SASP) of the epithelium impairs the microenvironment and affects the function of neighboring cells in a paracrine manner. Thus, the otherwise quiescent, healthy endothelium, which typically provides an efficient barrier to liquids and cell extravasation, becomes activated in response to certain epithelial-derived SASP factors (e.g., the chemokine MCP-1/CCL2). The resulting increased endothelial permeability causes leakage of bloodstream components into the lung interstitium, thereby fostering inflammation and/or metastais formation (acute reactions) (Hansel et al., 2021; Klein et al., 2017; Wiesemann et al., 2019). These effects could be efficiently reduced, thus protecting the endothelial compartment, when either induced epithelial senescence is limited or associated SASP factors are reduced (Hansel et al., 2021; Wiesemann et al., 2019). Supporting evidence arose from *in vitro* studies showing that radiation increases the permeability of the endothelial barrier by reducing the levels of proteins required for endothelial cell–cell contacts. Endothelial cell loss, contributing to long-term complications, was observed when irradiated, DNA-damaged endothelial cells began to proliferate following SASP factor stimulation (Wiesemann et al., 2019), a well-known phenomenon where radiation-induced endothelial changes were sustained and even progressive from months to years after the initial damage (late effects) (Satyamitra et al., 2016).

In addition to extrinsic stimulation, endothelial activation can also occur “from within” the endothelial cells due to intrinsic stimuli, e.g., following genotoxic stress. Genotoxic stress induced by radiation treatment, for example, can increase the production of ROS in endothelial cells, ultimately leading to DNA, mitochondrial, and/or plasma membrane damage, which results in severe endothelial activation. The induced expression of adhesion molecules and the release of inflammatory cytokines cause a similar (indistinguishable) activated endothelial phenotype in the irradiated endothelium as is caused by circulating factors (e.g., TNF-alpha and IL-6) or shear stress, the latter specifically due to turbulent blood flow. Thus, genotoxic stress-induced endothelial damage can lead to endothelial activation, followed by endothelial dysfunction and potential endothelial cell loss, contributing to severe adverse effects on the lungs (Wiesemann et al., 2019; Sharma and Himburg, 2022). This progression, initiated by intrinsic endothelial cell damage, might not be reversible. Endothelial activation, therefore, leads to changes in endothelial surface molecules, with the resulting functional alterations being expected to be reversible upon return to quiescence once the activating factors are removed.

## Sustained endothelial activation, dysfunction, and cell loss

Endothelial activation should be distinguished from endothelial dysfunction or cell damage, which may result from prolonged activation and can even progress to endothelial cell death (Klein, 2018; Butcher and Warner-Lambert/Parke-Davis Award lecture, 1990; Hunt and Jurd, 1998). Sustained endothelial activation has also been observed in lung injury, particularly during the development of pulmonary fibrosis (Fließer et al., 2024; Raslan et al., 2024). In fibrotic regions, a reduced barrier strength of the endothelium was found, as well as an increased sensitivity of the fibrotic endothelial cells to TNF-alpha or IFN-gamma, which in turn was associated with elevated immune cell adhesion and was based on an increase in the integrity markers PECAM-1/CD31, VE-CAD, thrombomodulin, and the VEGF receptor 2 (VEGFR-2/KDR) in different endothelial subpopulations (Fließer et al., 2024). It has been shown that a convergent signaling axis between the transcriptional regulator YAP and the tropomyosin-related kinase receptor B (TrkB) acts as a putative regulatory hub in persistently activated pulmonary endothelial cells (Raslan et al., 2024). A modulation of this axis, e.g., by brain-derived neurotrophic factor (BDNF), a ligand available from regenerating alveolar type I cells that act on the TrkB receptor of activated endothelial cells, could in turn promote endothelial morphogenesis (Raslan et al., 2024). Likewise, the transcriptional regulator FOXF1, a member of the forkhead box (FOX) family of transcription factors that is highly enriched in lung endothelial cells compared to endothelial cells of other organs, turned out to be a critical regulator of the transition from normal to fibrosis-associated endothelial cells (Bian et al., 2023). Decreases in endothelial FOXF1 in the course of pulmonary fibrosis was accompanied by increased endothelial permeability that contributed to aberrant inflammation and increased fibrosis. Therefore, restoring endothelial cell homeostasis and function, i.e., normalizing the pulmonary vascular bed, could represent an innovative therapeutic option for fibrotic lung diseases. An excellent review summarizing the transcriptional factors that mediate the transition from homeostasis to an activated endothelial cell state and the pathogenesis of (lung) diseases and which could serve as future therapeutic targets has just been published (Fließer et al., 2025).

Endothelial dysfunction is a pathological condition of the endothelium that encompasses a broader spectrum of phenotypes associated with heterogeneous changes in vascular permeability and inflammation, as well as vasoconstriction and thrombosis, thus leading to the loss of endothelial homeostatic functions (Alexander et al., 2021; Naderi-Meshkin and Setyaningsih, 2024). The timing of endothelial cell dysfunction turned out to be crucial, with endothelial activation and thus increased expression of adhesion molecules and increased vascular permeability being among the early events. Accordingly, markers associated with endothelial dysfunction enabling early diagnosis of the disease include acute-phase cytokines (such as IL-6, IL-1, and TNF-alpha) and adhesion molecules, particularly ICAM-1, VCAM-1, and E-selectin, which are involved in the adherence of inflammatory cells. Of note, similar pattern of cytokines and markers was also found for COVID-19 and septic patients, highlighting the importance of endothelial dysfunction here and further suggesting that the endothelium should be further evaluated as a therapeutic target for the disease (Libby, 2024; Xu et al., 2023; Hokama et al., 2022). Within sustained chronic activation and thus endothelial dysfunction (but also within acute activating conditions), the nuclear transcription factors NF-κB should be considered as key regulator of most cytokines and adhesion molecules induced in the endothelial cells (Guo et al., 2024; Hallahan et al., 1998; Poulos et al., 2016; Sonveaux et al., 2017). *Among the various cytokines,* TNF-alpha and thrombin have been shown to be the most potent activators of NF-κB signaling in endothelial cells (Soh et al., 2010; Mussbacher et al., 2019). TNF-alpha binds to its receptors (TNFR-1 and TNFR-2), mediating the plasma membrane assembly of TNFR1-RIP-TRADD-TRAF2 complexes (Guo et al., 2024). As a specific binding partner of TNFR1, epidermal growth factor receptor (EGFR) activation has been shown to mediate enhanced recruitment of TNFR-associated factor 2 (TRAF2) to the TNFR1 complex and to increase phosphorylation of receptor-interacting protein 1 (RIP1) in endothelial cells, ultimately increasing NF-κB/MAPK-mediated inflammation (Zhang et al., 2023). MMPs, for example, are targets of NF-κB signaling and promote transendothelial extravasation as well as shaping of the microenvironment (John and Tuszynski, 2001) and even impact on mitochondrial dynamics in endothelial cells (Herke et al., 2020). Protease-activated receptor 1 (PAR1) is the predominant thrombin receptor in endothelial cells (van den Eshof et al., 2017). Both ligand-receptor interactions downstream induce NF-κB signaling *via* activation of the IKKα/IKKβ/NEMO (IKKγ) complex, with synergistic effects (Tiruppathi et al., 2001). In addition to NF-κB-driven activation and resulting vascular dysfunction, the local stress-response processes in endothelial cells generally depend on the function of endothelial Weibel–Palade bodies, which are members of the family of lysosome-related organelles, harbouring proteins involved in inflammation and thrombosis such as coagulation factor VIII, von Willebrand factor, or P-selectin (Goligorsky et al., 2009). Following signal transduction from ligands as diverse as thrombin, histamine, growth factors (e.g., VEGF), and superoxide anions, exocytosis of Weibel–Palade bodies occurs, with membrane fusion and content release (Mussbacher et al., 2019; Goligorsky et al., 2009). Osteoprotegerin (OPG), a member of the TNF receptor superfamily, is a receptor activator of nuclear factor kappa-B ligand (RANKL), and TRAIL (TNF-related apoptosis-inducing ligand) mediates endothelial cell apoptosis inhibition by inhibiting TRAILR1/2 signaling (Rochette et al., 2018). At the same time, OPG has been shown to induce vascular dysfunction by promoting reactive ROS production (Le et al., 2022). ROS, in turn, are known to directly activate redox-sensitive transcription factors such as nuclear factor (erythroid-derived 2)-like 2 (NRF2) and activator protein 1 (AP-1) (Tiruppathi et al., 2001; Goligorsky et al., 2009).

Concerning the (chronic) endothelial activation-dysfunction interconnection, it has been mechanistically revealed that endothelium-derived NO generally prevents endothelial activation prior to endothelial dysfunction through inhibition of the transcription factor NF-κB (Liao, 2013; Baeuerle, 1998). Accordingly, endothelial dysfunction has been defined as the decreased synthesis, release, and/or activity of endothelium-derived NO (Liao, 2013), following the observation that eNOS inhibitor treatment increases the number of adherent and emigrated leukocytes to mesenteric venules (Kubes et al., 1991). Endothelial cells usually utilize NO production to regulate vascular tension *via* vasoconstriction and vasodilation to meet cellular oxygen demands. Increased NO levels lead to elevated cGMP and cAMP levels, which activate cellular kinase cascades, such as protein kinase A (PKA) or protein kinase G (PKG), leading to smooth muscle relaxation. NO also increases platelet cAMP levels and serves as an important platelet aggregation inhibitor, a mechanism that is impaired during endothelial dysfunction. The shift from a non-adhesive endothelial surface, which supports unrestricted blood flow by inhibiting platelet aggregation and fibrin formation, to an adhesive surface that captures circulating blood cells following endothelial activation (Naß et al., 2021) also causes damage to the coagulation system (Qiao et al., 2024). Endothelial cells regulate the balance between coagulation and fibrinolysis through the release of tissue factor pathway inhibitor (TFPI), the activated protein C (APC) system, expression of thrombomodulin, and the synthesis and release of tissue plasminogen activator (t-PA). Activated endothelial cells and leukocytes release tissue factor (also called coagulation factor III) into the bloodstream to trigger acute intravascular thrombus formation following interaction with other coagulation factors, mainly factor VII. Furtheron, downregulation of the surface receptor thrombomodulin, an anticoagulant cofactor, increases free thrombin and activates platelets, creating a pro-thrombotic state (Giri et al., 2024). Similarly, fibrinolysis inhibitors, such as plasminogen activator inhibitor-1 (PAI-1) and thrombin-activatable fibrinolysis inhibitor (TAFI), can be detected following prolonged vascular stress in septic conditions, which can lead to abnormal blood clotting throughout the body’s blood vessels, resulting in disseminated intravascular coagulation (Papageorgiou et al., 2018). Endothelial dysfunction further impacts endothelial morphology, including endothelial hypertrophy associated with the reorganization of actin filaments and detachment from the basement membrane, ultimately causing vascular constrictions, thrombosis due to hypercoagulation, platelet aggregation, and rupture of microvascular walls (Fajardo et al., 2008). The latter contributes to an increase in chronic permeability.

Beside the reduced production of NO, endothelial dysfunction has been linked to mitochondrial damage and dysfunction mediated by the (chronic) overproduction of ROS or impaired energy production (Wang et al., 2023). The imbalance of NO and ROS, generally promoting endothelial dysfunction can be roughly summarized as oxidative stress (Higashi et al., 2014; Lugrin et al., 2014). The formation of ROS is actually an inevitable consequence of living in an oxygen-rich environment. However, excessive formation of oxygen radicals under pathological conditions can cause severe damage to the pulmonary endothelium, which has been summarized in a very elegant review (Kellner et al., 2017). In addition to endothelial cells, vascular wall cells and interstitial fibroblasts, immune cells such as neutrophils, eosinophils and alveolar macrophages as well as alveolar epithelial cells are the cellular ROS and also RNS generators. On molecular level, activation of nicotinamide–adenine dinucleotide phosphate (NADPH) oxidase, xanthine oxidase, cyclooxygenase, and uncoupled endothelial nitric oxide synthase (eNOS) and mitochondrial electron transport lead to an increase in ROS production, particularly in production of the superoxide radical (O_2_^.−^) (Higashi et al., 2014; Kellner et al., 2017). Inactivation of the antioxidant system (e.g., superoxide dismutase, glutathione peroxidase and catalase), and thus a reduction in ROS degradation could further contribute to enhanced levels. The ROS dependent expression of adhesion molecules by endothelial cells in turn fosters immune cell recruitment to the site of inflammation that themselves generate high levels of ROS thereby contributing to the oxidative stress levels (Kellner et al., 2017). The simultaneous generation of NO, a highly reactive, gaseous free radical, by eNOS, can locally interact with O_2_^.−^ to make peroxynitrite (ONOO−), a RNS. eNOS is the main source of NO with strong vasodilatory, anti-inflammatory, and antioxidant properties in healthy (pulmonary) endothelium, which is constitutively expressed at low levels (in the picomole range) and requires increased intracellular Ca2+ for activation. Inducible NOS (iNOS), on the other hand, acts calcium-independent and is the main NOS producer in response to conditions of stress and inflammation (Lowry et al., 2013). Furthermore, iNOS was systematically localized predominantly in the lungs (Pitt and Croix, 2002). iNOS was shown to be widely distributed in the airway epithelium and vascular smooth muscles, and was especially found to be expressed by expressed by inflammatory cells such as neutrophils and macrophages (Geller and Billiar, 1998; Golden et al., 2021). Furthermore, immunoreactive iNOS was found within the lung endothelium in areas of chronic inflammation and even seems to be constitutively expressed in upper and lower airway epithelium in normal lung tissue. (Kobzik et al., 1993; Pitt and Croix, 2002; Uetani et al., 2001; Farahani et al., 2025). Generally, NOS catalyze the production of NO and L-citrulline from L-arginine and O_2_, using electrons donated from NADPH, a process termed coupled eNOS activity (Cyr et al., 2020). In the uncoupled process, electrons are passed directly to O_2_, generating superoxide, which can ultimately combine with locally produced NO to generate peroxynitrite. Generated peroxynitrite then induces nitrasative stress on cells by nitrating proteins and thus, together with the promotion of oxidation of biomolecules *via* secondary oxidants (e.g., nitrogen dioxide radical (·NO_2_) and hydroxyl radical (·OH)) alters signaling pathways thereby promoting tissue damage (Beckman and Koppenol, 1996; Bartesaghi and Radi, 2017). Certain growth factor in addition, for example, TNF-alpha, were shown to induce a peroxynitrite-dependent increase in pulmonary endothelial permeability that is associated with generation of nitrated beta-actin, which alters actin-polymerization dynamics and thus ultimately barrier function (Neumann et al., 2006). Depending on production rates, endogenous antioxidant levels and exposure time, peroxynitrite can even induce both cellular apoptosis and necrosis (Szabó et al., 2007). Within that scenario, mitochondrial depolarization and loss of redox capacity have been described as (early) targets for both nitric oxide and peroxynitrite-mediated cellular effects. Herein, nitric oxide primarily inhibits cytochrome oxidase (complex IV) and potential interactis with iron sulfur proteins, whereas peroxynitrite inhibits complexes I-III finally resulting in the collapse of the mitochondrial membrane potential and the in a severe decline in energy production (Gow et al., 1998; Szabo et al., 2007; Haddad et al., 1994). In contrast, endothelial cells have been described to be equipped with fewer mitochondrial organells (Parra-Bonilla et al., 2010), which coincides with the observation that concerning the metabolsim glycolytic pathways have primacy over mitochondrial respiration (Stevens et al., 2021; Li et al., 2019). This means that pulmonary lung endothelial cells produce their energy, i.e., ATP, primarily through aerobic glycolysis, despite being exposed to higher oxygen concentrations than other cells. This metabolic strategy is thought to facilitate oxygen diffusion to neighboring cells by minimizing their own oxygen consumption and reducing the formation of ROS (Mora Massad et al., 2025; Pi et al., 2018; Leung and Shi, 2022). Although impaired glycolysis under pathological conditions may lead to the activation of alternative metabolic pathways to maintain energy balance, this would be accompanied by increased oxidative stress, which can then lead to dysfunction and ultimately the death of endothelial cells (Mora Massad et al., 2025; Pi et al., 2018; Leung and Shi, 2022). In addition, lung microvascular endothelial cells appear to have a predominately socalled fission phenotype of the mitochondria, which make emdothelial cells more sensitive to metabolic disturbances under diseased conditions (Pokharel et al., 2024).

Severe endothelial cell stress, caused by vascular dysfunction or direct endothelial cell damage, particularly in response to genotoxic stress, promotes endothelial cell death. Induced (sublethal) DNA damage and/or DNA repair deficiencies may contribute to the activated endothelial cell state through the activation of the genotoxic stress-induced NF-κB signaling machinery (Hellweg, 2015; Liu et al., 2017). Intrinsic endothelial activation following oxidative stress, namely, the activation of the endothelium to a pro-inflammatory state in the absence of typical endogenous factors or pathogens, is known as “sterile inflammation” (Baselet et al., 2019). Altered metabolic products of dying or stressed (neighboring) cells can also serve as danger signals to activate innate immune cells. When released DAMPs bind to endothelial cells, they subsequently upregulate pro-inflammatory signaling pathways, leading to NF-κB, MAPK, and interferon regulatory factor 3 (IRF3) signaling (Liu et al., 2017; Zindel et al., 2020). Severe endothelial cell loss is hypothesized to occur as a long-term complication, as irradiated, DNA-damaged, and further activated endothelial cells start to proliferate, which subsequently leads to apoptosis (Klein et al., 2017; Wiesemann et al., 2019). Endothelial apoptotic cell death following severe endothelial stress can be mediated by either p53 or the p53-independent sphingomyelin ceramide pathway (Chopra et al., 2022). The activation of p53 *via* phosphorylation (e.g., following DNA damage induction) triggers endothelial cell cycle arrest. Insufficient DNA damage repair initiates apoptotic cell death *via* the intrinsic cytochrome c-mediated mitochondrial pathway, the extrinsic TNF death receptor pathway, or the activation of the sphingomyelin ceramide pathway (Venkatesulu et al., 2018). Endothelial cell death has been closely linked to acid sphingomyelinase (ASMase)-induced ceramide production, which occurs through the proteolytic cleavage of sphingomyelin present in the outer leaflet of the plasma membrane. The resulting plasma membrane alterations and generated lipid rafts cause clustering of receptors, including death receptors (Hernández-Corbacho et al., 2015; Pilátová et al., 2023). Mechanistically, induced ASMase activity and subsequent ceramide generation foster the formation of large lipid platforms, ultimately altering p38 mitogen-activated protein kinase (MAPK)/heat-shock protein 27 (HSP27)/AKT (protein kinase B, PKB) signaling to enhance apoptotic processes (Wiesemann et al., 2019).

## Conclusion

As part of normal tissue homeostasis, the initiation of endothelial activation directs immune cells to sites of tissue injury, mediating protective effects of endogenous anti-inflammatory systems. The main feature of the activation and subsequent dysfunction of the pulmonary endothelium is increased permeability, which leads to the development of a pro-inflammatory phenotype characterized by increased expression of adhesion molecules to recruit inflammatory cells, by activation of pro-inflammatory transcription factors, and by release of inflammatory mediators. These changes can result in vascular leakage and edema formation. Additionally, increased oxidative stress, an altered balance between vasoconstriction and vasodilation, and a thrombotic phenotype have been implicated in the loss of endothelial homeostatic function. Depending on the nature, extent, duration, and combination of pro-inflammatory stimuli, this physiological activation can progress to detrimental pathological activation, resulting in endothelial dysfunction. Further progression to the death of pulmonary endothelial cells, along with dysfunction or even death of alveolar epithelial cells, can lead to the disruption of the alveolar-capillary barrier, a process that characterizes ALI and its clinical manifestation, ARDS. Although significant progress has been made in understanding pulmonary endothelial dysfunction, the triggers, mechanisms, and consequences of a dysfunctional endothelium in acute and chronic lung diseases are still not fully understood. A better understanding of these key aspects will help identify new disease biomarkers and/or therapeutic targets to maintain homeostasis in response to injury and disease.
